# Biphalin—A Potent Opioid Agonist—As a Panacea for Opioid System-Dependent Pathophysiological Diseases? †

**DOI:** 10.3390/ijms222111347

**Published:** 2021-10-21

**Authors:** Patrycja Redkiewicz, Jolanta Dyniewicz, Aleksandra Misicka

**Affiliations:** 1 Department of Neuropeptides, Mossakowski Medical Research Institute Polish Academy of Sciences, 02106 Warsaw, Poland; misicka@chem.uw.edu.pl; 2 Faculty of Chemistry, University of Warsaw, 02093 Warsaw, Poland

**Keywords:** biphalin, opioid system, opioid receptors agonist, multidirectional, opioid peptides, analgesic, non-analgesic, pain, immunomodulatory agent, wound healing

## Abstract

Biphalin, one of the opioid agonists, is a dimeric analog of enkephalin with a high affinity for opioid receptors. Opioid receptors are widespread in the central nervous system and in peripheral neuronal and non-neuronal tissues. Hence, these receptors and their agonists, which play an important role in pain blocking, may also be involved in the regulation of other physiological functions. Biphalin was designed and synthesized in 1982 by Lipkowski as an analgesic peptide. Extensive further research in various laboratories on the antinociceptive effects of biphalin has shown its excellent properties. It has been demonstrated that biphalin exhibits an analgesic effect in acute, neuropathic, and chronic animal pain models, and is 1000 times more potent than morphine when administered intrathecally. In the course of the broad conducted research devoted primarily to the antinociceptive effect of this compound, it has been found that biphalin may also potentially participate in the regulation of other opioid system-dependent functions. Nearly 40 years of research on the properties of biphalin have shown that it may play a beneficial role as an antiviral, antiproliferative, anti-inflammatory, and neuroprotective agent, and may also affect many physiological functions. This integral review analyzes the literature on the multidirectional biological effects of biphalin and its potential in the treatment of many opioid system-dependent pathophysiological diseases.

## 1. Introduction

The opioid system consists of three classical opioid receptors—termed μ (MOR), δ (DOR), and κ (KOR)—a non-classical nociceptin receptor (NOR), and endogenous opioid peptides (dynorphins, enkephalins, endorphins, and endomorphins). Opioid receptors are widely distributed throughout the human body in the central and peripheral nervous systems [1]. In peripheral tissues, opioid receptors are expressed in various cells in both neuronal and non-neuronal tissues, including neuroendocrine, immune, and ectodermal cells [2]. Opioid peptides and their receptors play an important role in many physiological functions: mainly pain and analgesia, but also stress and social status; tolerance and dependence; mental illness and mood; general activity and locomotion; gastrointestinal, renal, and hepatic function; cardiovascular responses; respiration and thermoregulation; immunological responses; seizures and neurological disorders; electrical-related activity and neurophysiology; and others [3]. Every year, more than a thousand papers devoted to the opioid system, its functions, and its ligands are published [4,5,6,7,8].

Endogenous opioid peptides, which have been the subject of intensive research for several decades, are the basis for the creation of new opioid peptides, more stable to enzymatic degradation and causing fewer side effects than commonly used analgesic drugs such as morphine or fentanyl. The development of new opioid drugs should take into account the multidirectional action of the opioid system. One example of an opioid ligand with multidirectional activity is biphalin.

Biphalin was first synthesized and described by Andrzej W. Lipkowski in 1982 [9]. Almost 40 years after this first publication, biphalin is a well-recognized, commercially available opioid agonist. The reviews published so far have been devoted primarily to its structure–activity relationship and its numerous analogs [10,11]. In this review, we focus on the multidirectional activity of biphalin.

**Figure 1:**
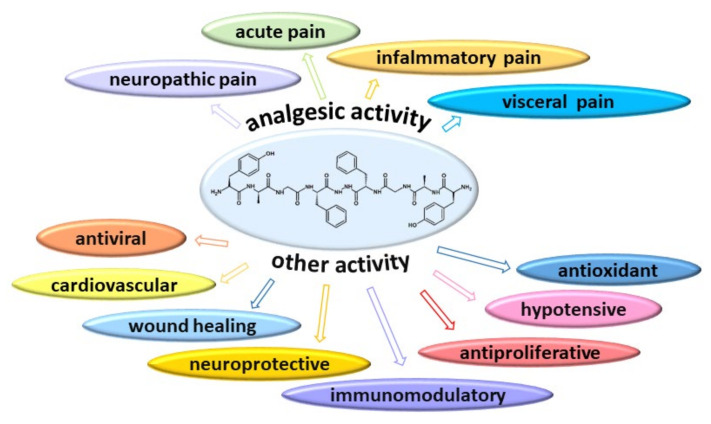
Schematic diagram of the multidirectional activity of biphalin.

Primarily, biphalin was designed as an analgesic peptide. Extensive research on its antinociceptive effect has shown its excellent properties in acute, neuropathic, and chronic pain. In the course of extensive research devoted primarily to the antinociceptive effect of this compound, it has been found that biphalin may also potentially participate in the regulation of other opioid-dependent functions. Biphalin as a non-selective ligand of opioid receptors was studied as an antiproliferative, antiviral, immunomodulatory, neuroprotective, wound healing-improving, hypotensive, antioxidative, respiratory agent (Figure 1).

## 2. Chemistry and Pharmacology of Biphalin

**Figure 2:**
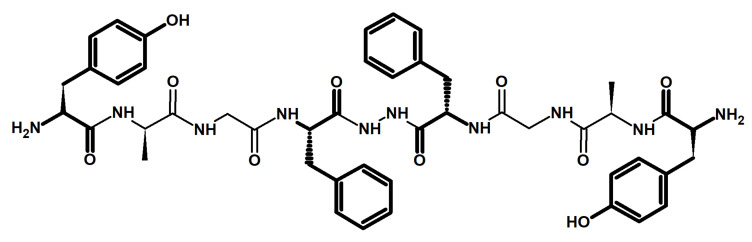
Biphalin structure with marked critical fragments.

Biphalin is a symmetrical dimeric analog of enkephalin consisting of two identical tetrapeptides (N-terminal fragments of enkephalin) linked tail-to-tail by a hydrazide bridge (H-Tyr-D-Ala-Gly-Phe-NH-NH-Phe′-Gly′-D-Ala′-Tyr′-H) (Figure 2). The main approach to the synthesis of biphalin is still the solution-phase peptide synthesis strategy [12]. It has been shown that the N-terminal tyrosine is critical for the high affinity of biphalin to the opioid receptors and for pharmacological activity. The tetrapeptide fragments of biphalin are very flexible, whereas the hydrazide bride is very rigid. The X-ray structure of the sulfate salt of biphalin shows topographical differences between the two identical connected peptide fragments presented in the molecule [13]. The structural analysis shows that the two carbonyl groups of phenylalanine residues and the two nitrogen atoms of hydrazide exist in two forms. The first of them has a planar location of all elements of the bridge [14], and in the second, the two planar halves of the bridge are located at approximately 60° of torsion across the N–N bond [13]. In the second conformation, two tetrapeptide parts are not conformationally equivalent. The amino acid residues 1–4 in biphalin are well aligned with the δ-selective opioid peptide DADLE (H-Tyr-D-Ala-Gly-Phe-D-Leu-OH), whereas residues 5–8 can be fit to the μ-selective peptide D-TIPP-NH2 (H-Tyr-Tic-Phe-Phe-NH2) [13].

Initially, biphalin was reported as a very potent inhibitor of electrically induced contractions of guinea pig ileum, as producing a strong analgesic effect when administered intraperitoneally in mice, and as potentially interacting with μ opioid receptors [9]. Moreover, biphalin’s hypothermic effect on mice suggested activity through MOR [15]. The suggested affinity to opioid receptors was verified and confirmed by Lipkowski et al. in 1987 [16]. The experimental studies have shown that biphalin is a nonselective ligand with a very high binding affinity for opioid receptors: Ki = 12 ± 2 and 4.6 ± 0.2 nM for MOR and DOR respectively, and a much lower binding affinity for the KOR receptor Ki = 270 ± 15 nM. The high affinity was confirmed by multiple binding assays in various laboratories, and biphalin affinity was found in the following ranges: for MOR Ki = 0.19 − 12 nM, for DOR Ki = 1.04 − 46.5 nM, and for KOR Ki = 270 − 283 nM (Table 1).

Further studies have shown that only one tetrapeptide with a hydrazide bridge and phenylalanine residue from the other tetrapeptide is responsible for the biological properties of biphalin [19]. The results were used to design and then synthesize a series of truncated biphalin analogs with a shortened second arm or with its replacement with another pharmacophore [25,26,27,28]. It was shown that even the hydrazide of tetrapeptide (H-Tyr-D-Ala-Gly-Phe-NH-NH_2_) exhibited a good affinity for MOR, similar to the affinity of biphalin, but this compound had a 100 times lower affinity for DOR. Replacing the second phenylalanine (Phe’) with a non-aromatic, lipophilic, or other aromatic amino acid did not significantly change the binding affinity to MOR or DOR [25]. Biphalin-based heterobivalent ligands for various receptors, such as cholecystokinin, neurokinin-1, melanocortin, and calcium channel blockers have also shown promise in the field of analgesics due to the synergistic action of many of the targets involved [26,27,28,29,30,31,32,33]. The efficacy of the opioid agonist in the functional characterization of biphalin at the MOR and DOR receptors was examined by a [^35^S] GTPγS binding assay (Table 2). It was found that biphalin effectively stimulates the G-proteins, which indicate its agonist properties [34]. On the other hand, biphalin demonstrated lower efficacy compared to deltorphin II (a selective DOR agonist) and total receptor occupancy was required to obtain the maximal response. It was shown that biphalin does not efficiently stimulate G proteins through DOR, and it was suggested that the antinociceptive response of biphalin may be partially attributed to MOR activation [24].

The binding properties of biphalin correlate with the bioassay results performed on isolated tissue: mouse vas defense (MVD) and guinea pig ileum (GPI) [25] (Table 3). Biphalin is almost 90 times more potent than Met-enkephalin and 11.7 times more potent than D-Ala^2^-Met-enkephalinamide in the guinea-pig ileum (GPI) in vitro test [9].

The pronounced binding affinity of biphalin to opioid receptors is reflected in its analgesic properties. Lipkowski et al. suggested that the key role in the analgesic effect of biphalin is played by its ability to cross the blood–brain barrier (BBB), but in 1988, Kamei and Kasua predicated that it is unclear whether biphalin can easily penetrate the BBB [37]. An experiment on bovine brain microvessel endothelial cell (BMEC) monolayers, and the in vivo BBB study on mice, demonstrated the ability of biphalin to cross these cells and suggested that factors other than lipophilicity may play a role in BMEC passage [38]. Further studies on the ability of biphalin to penetrate the BBB exhibited how both biphalin and its chlorohalogenated analogs can enter the CNS through both the blood–brain and blood–cerebrospinal fluid barriers and exhibit biological stability in rat brain homogenates after an in situ brain perfusion [39]. To explain biphalin’s potency, regional brain and spinal cord distribution studies of [^125^I-Tyr1]-biphalin have been performed [40]. The role of a large neutral amino acid (LNAA) carrier in biphalin’s transportation through the BBB was confirmed. The stability study of biphalin in rat brains reflects the stability measured in the in vitro brain membrane studies using 15% mouse brain membranes, showing a metabolic half-life of 173 min [41]. Albekairi et al. presented studies on the role of organic anion transporting polypeptide 1 (OATP1) in the transport mechanism of biphalin across the BBB during an ischemic stroke in an in vitro model using human induced pluripotent stem cell-differentiated brain microvascular endothelial cells (iPSC-BMECs) [42]. The effect of oxygen-glucose deprivation (OGD) and OGD-reperfusion (OGD/R) on the uptake of biphalin by iPSC-BMECs was also studied. The results partially confirmed the role of OATP1 transporters in the uptake of biphalin by brain endothelial cells in an in vitro model of a stroke (OGD-reperfusion). Moreover, the BBB permeability of biphalin was tested in an in vivo model of allergic encephalomyelitis (EAE). This condition is known to increase the permeability of the BBB. The antinociceptive potency of intravenously injected biphalin proportionally increased according to the magnitude of systemic inflammation and correlated well with the progression of EAE [43]. Romanowski’s studies on the permeation of biphalin across model membranes, composed of neutral phospholipids and cholesterol, indicated that permeation is controlled by diffusion and depends on the water-membrane partition coefficient [44]. It has been proposed that the interaction of phenylalanine and tyrosine aromatic rings can promote the peptide’s ability to penetrate the model membranes, stabilizing the more compact structure of biphalin by minimizing the number of hydrogen bonds with water [45]. Further studies showed that anionic lipids, naturally present in cell membranes, may reduce the transportation of some peptides such as biphalin across the membrane [44]. The mechanism of action of biphalin was also studied by electrophysiological analyses of its effects on the action potential duration (APD) of nociceptive types of sensory dorsal root ganglion (DRG) neurons in culture [46]. Extensive studies on the ability of biphalin to cross the BBB showed, unequivocally, that it passes the barrier only to a small extent [38,47].

## 3. Biphalin as an Analgesic Agent (In Vivo Studies)

Since the first publication on biphalin, its analgesic effects have been tested in many studies using different animal pain models (acute, chronic, visceral), different routes of administration (intraperitoneally (i.p.), subcutaneously (s.c.), intravenously (i.v.), intrathecally (i.t.), intracerebroventricularly (i.c.v.), intramuscularly (i.m.), and intracutaneously (i.c.)), and various assays (tail-flick, hot plate, cold plate, Hargreaves, von Frey, formalin, mustard oil) (Table 4 and Table 5). Different assays and different routes of administration allow for the research of biphalin’s ability to cross barriers and its ability to act via the central or peripheral nervous systems. It is considered that responses in the hot-plate and paw withdrawal test are supraspinal, whereas the tail-flick test is used as a spinal analgesic assay. The formalin test is an important animal model in the study of acute, long-lasting pain, whereas mustard oil inflicts inflammatory pain. In each of these studies, biphalin showed an analgesic effect, which proves its versatility and uniqueness among other known analgesic compounds.

The first research on the analgesic activity of biphalin demonstrated that it possesses a very high potency and produced statistically significant analgesia at doses 10 and 20 mg per kg of body weight 30 and 60 min after intraperitoneal injection, respectively (Table 4) [9]. The biphalin increased the response latency by 82.3% compared to an equimolar dose of morphine hydrochloride. The analgesic effect was antagonized by a naloxone pretreatment (6.4 mg of naloxone hydrochloride per kg of body weight) which suggests that biphalin acts via opioid receptors [9].

Further studies on the antinociception of biphalin showed excellent activity after intrathecal administration [16]. The obtained results suggested that the DOR is involved in spinal nociceptive modulation in mice.

The other studies showed that biphalin’s antinociceptive potency was about two-fold higher than the potency of equimolar doses of morphine [37]. Biphalin and morphine produced a dose-dependent decrease in the frequency of respiration and in tidal volume. For biphalin, the effects were significantly weaker than for morphine. The antitussive effect of biphalin is antagonized by naloxone, indicating that opioid receptors are involved.

The analgesic activity of biphalin was also tested after subcutaneous (s.c.), intravenous (i.v.), and intrathecal (i.t.) administration [48]. The effect was assessed in comparison to morphine in rats using tail-flick and tail-pinch tests (Table 4). Biphalin administered s.c. showed negligible analgesic activity, but after i.v. injection produced significant analgesia, though weaker than morphine administered via this route. After intrathecal administration, biphalin was more potent than morphine. These results indicated that biphalin has an intrinsic activity that is compromised by enzymatic degradation or redistribution in the periphery. After the i.t. administration of biphalin in two out of twelve rats, rigidity occurred, i.e., a marked increase in muscle tone involving the whole body, becoming apparent 15 min after injection. This rigidity began to decrease after 60 min, and muscle tone returned to normal after 120 min. No evidence of any flaccid paralysis was observed for biphalin after this route of administration. Several rats displayed transient rigidity after the i.v. administration of biphalin. In contrast to the i.t.-administered group, the increase in muscle tone became apparent immediately following injection, began to wane after 10 min, and returned to normal after 45 min. Rigidity was not observed in rats given morphine by this route. This activity of biphalin is explained by the fact that biphalin’s structure confers some resistance to enzymatic breakdown due to two factors: first, D-Ala replaces L-Gly at position 2; and second, the presence of the hydrazide bridge replaces the carboxyl groups.

The analgesic activity of biphalin was also tested after an acute burn injury (Table 4) [49]. The drugs morphine (a predominantly MOR agonist), biphalin (a predominantly MOR and DOR agonist), and U50,488H (a predominantly KOR agonist) were administered intravenously. The authors concluded that analgesic potency after a burn injury tends to augment stress-induced analgesia and is relatively independent of receptor selectivity. The release of beta-endorphins and corticosterone that follows acutely after burn injuries is not prevented by prior administration of analgesic doses of opioids and may play a role in the enhanced potency of the analgesia.

The effect of the co-administration of morphine-3-glucuronide (M3G) upon opioid antinociception produced by biphalin was also tested [50] (Table 4) and it was found to have no effect at any time on biphalin’s analgesia. Another study showed that the co-administration of a substance P antagonist with biphalin significantly enhanced and prolonged the antinociceptive effect of biphalin [51].

Horan et al. published data on biphalin’s antinociceptive profile in mice and studied the gastrointestinal effects, penetration to the brain, biological stability, and physical dependence profiles of biphalin (Table 4) [47]. The results of this study indicated that biphalin acts through opioid μ1, μ, and δ2 receptors and may produce antinociception via a complex and unusual mechanism. Furthermore, biphalin appears to produce fewer side effects (e.g., gastrointestinal effects and physical dependence) than traditional opioids such as morphine while still providing substantial antinociceptive action. It has been shown that the potency of biphalin is almost seven-fold greater than that of the alkaloid opioid agonist etorphine, and 7000-fold greater than that of morphine. Moreover, this study pointed out that after i.p. administration, the penetration of biphalin into the brain is limited (approximately 0.05% after 20 min).

Further data on antinociception after the intrathecal administration of biphalin were published in 2005 (Table 4) [53]. This result demonstrated that biphalin produces intense analgesia with a wide therapeutic window. The administration of 20 nmol of biphalin produced transient muscle rigidity but no respiratory depression. The muscle rigidity lasted up to 3 h and then returned to normal, but the antinociception persisted for several hours longer.

Another study on the antinociceptive potency of biphalin showed that the intrathecal co-administration of ketamine (an NMDA antagonist) with biphalin produced markedly greater antinociception than biphalin injected alone in acute thermal tail-flick tests in rats (Table 4) [54]. Rat i.t. pretreatment with naltrexone eliminated the antinociceptive effects. The administration of biphalin or of the mixture of biphalin and ketamine for ten consecutive days resulted in the development of antinociceptive tolerance. This tolerance developed more rapidly after biphalin–ketamine mixture administration.

The excellent analgesic activity of biphalin and its limited BBB permeability, which promotes peripheral analgesia, was the reason to test it in vivo in mice in a chronic cancer pain model using intravenous administration (Table 5) [21]. The effects were compared to the gold standard in chronic cancer pain treatment—morphine. The study showed that the participation of peripheral analgesia after biphalin usage is more robust than in animals receiving morphine. Dose-dependent analgesia in mice showed thermal hypersensitivity evoked by intraplantar B16F0 melanoma cell implantation. In the animal model of skin cancer pain, the mice developed robust thermal hypersensitivity restricted to the tumor-bearing paw that was correlated with tumor growth. Biphalin produced spinal analgesia without the development of spinal tolerance. The authors speculated that more robust activation of peripheral opioid receptors by biphalin might be attributed to either the increased affinity of biphalin at the MOR and DOR (and possibly KOR) receptors or its limited BBB permeability, due to the differential effectiveness of the transport mechanisms.

It was also demonstrated that biphalin was able to reduce formalin-induced pain behavior both in the early (acute pain) and in the late phase (inflammatory pain) stages of the test after s.c. administration (Table 5) [22].

In a preclinical model of neuropathic pain in rats, biphalin reduced pain-related behavior (Table 5) [56]. In CCI-exposed rats, biphalin attenuated the development of tactile and thermal hypersensitivity, as measured by von Frey and cold plate tests, respectively, as compared to the vehicle-treated CCI-exposed animals.

Another study showed that biphalin displays antinociceptive activity in an animal model of colitis induced by i.c. injection of trinitrobenzenesulfonic acid (TNBS) in the hot plate test. Moreover, biphalin injected i.p. and i.c. decreased the number of mustard oil-induced pain-related behaviors in TNBS-treated mice (Table 5) [57].

In further studies on biphalin, less liability for physical dependence on biphalin was found following chronic biphalin infusion in rats, which was different from that of morphine [58]. Only minor withdrawal signs were observed after chronic biphalin infusion in rats by a naloxone challenge, whereas in morphine-dependent rats, naloxone precipitated severe withdrawal signs. It was suggested that this effect could be the result of the difference between biphalin and morphine selectivity to opioid receptors and the interactions between the types of opioid receptors. It was found that biphalin is able to reverse the symptoms of pentazocine withdrawal, but not morphine withdrawal.

In conclusion, extensive studies on the antinociceptive potency of biphalin, performed in many different laboratories, demonstrated its excellent properties. The analgesic potency of biphalin was manifested in acute, neuropathic, and chronic pain. It was proven that biphalin acts in peripheral and CNS opioid receptors without substantial side effects in comparison to the gold standard pain treatment—morphine.

## 4. Other Activity of Biphalin

In the previous chapter, the results of the research on biphalin in terms of its analgesic effects were discussed. This chapter discusses studies that have shown that, in addition to its analgesic properties, biphalin may play a beneficial role as an antiviral, antiproliferative, immunomodulatory, neuroprotective, and antioxidative agent, and may also affect many physiological functions (Table 6).

### 4.1. Biphalin as an Antiviral and Antiproliferative Agent

Research into the anti-viral activity of biphalin was conducted by Tang et al. in 1998 [59]. The authors, using Mus dunni cells infected with Friend leukemia virus (FLV), a focus-forming assay (FFA), and a reverse transcriptase assay (RT), demonstrated that biphalin in a dose-dependent manner could play a beneficial role as a component in antiviral multidrug therapies (Table 6). The combination of biphalin with an inhibitor of reverse transcriptase (AZT, 3′azido-3′ deoxythymidine) allowed the reduction of the concentration of these two components to 15 to 30 μg/mL of biphalin and 0.5 ng/mL of AZT, giving a 50% inhibitory effect on FLV replication, which was more than the effect exerted by either biphalin or AZT used alone at this low concentration [59]. However, biphalin used alone at a 10^−6^ to 10^−8^ concentration in an RT assay diminished FLV reverse transcriptase activity as well. Next, in 2008, using the same assays, the same group confirmed the inhibitory effect of biphalin on the replication of FLV viruses. Additionally, a few experiments based on the combination of biphalin with AZT or/and splenocytes and/or INF-α showed that biphalin could improve the inhibitory effects of these components on the replication of FLV viruses by 30%, as compared with experiments where they were used alone [60].

The antiproliferative (anticancer) activity of biphalin was presented in 2010 by Lazarczyk et al. [61]. The authors studied the modulatory effect of biphalin and morphine on human glioblastoma T98G cell growth (Table 6). Their results showed that biphalin, compared with morphine, at doses of 10 μM and 40 μM, displayed an inhibitory effect on T98G cell proliferation. Additionally, a colony-forming assay demonstrated that prolonged—about 7 days—treatment of T98G cells with 40 μM of biphalin also limited the cells’ ability to form clusters. These results are promising and indicate that the use of biphalin in the chronic treatment of malignant glioma cancer, which is resistant to standard treatments using chemo- and radiotherapy, might limit tumor progression.

### 4.2. Biphalin as an Immunomodulatory Agent

In immune systems, opioid peptides seem to link neuroendocrine and immune systems to the control of immunological responses, in particular the modulation of inflammation (Table 6). There are several reports on the physiological and pathophysiological roles of endogenous and exogenous opioid peptides in the immune response [1,76]. The explanation of this role may lead to the development of new therapies using opioid peptides in the treatment of immune diseases.

Several independent groups focused on the immunomodulatory action of biphalin. (Table 6) The first, Mehrotra et al. [62], in an in vitro study, using several types of immune cells—lymphocyte T, NK cells, a suspension of human PMBCs, and mouse macrophage RAW 264.7—examined the immunomodulatory properties of biphalin. They demonstrated that biphalin, at a concentration of 10^−8^ or 10^−10^ M, with the optimal activity observed in a 72 h culture, stimulated the proliferation of the human T cells and significantly augmented human NK cell cytotoxicity. The authors supposed that this peptide probably released chemokine-like factors in the PMBC culture supernatants, which was observed as increasing the chemotaxis activity of monocytes. Next, in the same concentrations as above, biphalin and its analog, incubated with PMBCs, increased IL-2 production in comparison with PMBCs incubated alone. It marginally inhibited TNF-α production alone or in combination with the LPS stimulation of PBS cells and marginally inhibited NO production in RAW 264.7 cells after incubation with LPS. This study falls in line with previous work on the anti-inflammatory effects of enkephalin and demonstrates the immunomodulatory potential of biphalin [77,78].

Very interesting results associated with neuroinflammation are presented in a publication by Popiołek-Barczyk K. et al. [56]. They examined the effect of biphalin on neuropathy syndromes in a preclinical model of neuropathic pain in rats (CCI, chronic construction injury of the sciatic nerve) (Table 6). They also studied the effect of biphalin on the production of crucial pro- and anti-inflammatory factors, and members of the intracellular signaling pathway. This part of the study was conducted in vitro using a microglia cell culture prepared from cerebral cortices.

Firstly, they indicated that the intrathecal administration of biphalin at concentrations of 20, 200, and 1000 µM significantly attenuated the development of tactile hypersensitivity (measured by the von Frey test, 30 min after drug administration) and the development of thermal hypersensitivity (measured by the cold plate test, 35 min after drug administration) compared to the vehicle-treated CCI exposed animals. The in vitro studies indicated that biphalin significantly diminished the MOR receptor level as well as LPS-induced NF-kappaB, IkB, p-p38MAPK, and TRIF levels, and also pronociceptive mediators such as iNOS, IL-1 beta, and IL-18. In this case, the anti-inflammatory effect of biphalin was reversed by naloxone, which confirms that the activity of biphalin is dependent on the opioid receptors. Biphalin also reduced the levels of IL-6, IL-10, TNF-alfa, p-STAT3, and p-ERK1/2 and upregulated SOCS3, TLR4, and MyD88, but this effect was not reversed by naloxone.

Taken together, this study shows that, contrary to morphine [79,80,81,82,83], the analgesic effect of biphalin is correlated not only with the action of opioid receptors but also with diminished microglia-induced inflammation. Additionally, due to the lack of MOR receptors on microglia within the spinal cord [84], the studies of Popiołek-Barczyk K et al. [56] suggested that the analgesic effect of biphalin might also be mediated in an opioid receptor-independent manner.

In the gastrointestinal (GI) tract, opioid receptors are present in smooth muscle cells and at the terminals of sympathetic and sensory peripheral neurons. They are involved in the maintenance of homeostasis via the modulation of peristalsis, epithelial secretion, and fluid and electrolyte absorption, but may also be implicated in the pathophysiology of inflammatory and functional GI diseases. A large group of gastrointestinal disorders includes inflammatory bowel disease (IBD) and irritable bowel syndrome (IBS). Currently, no effective therapy for IBD or IBS is available. The activity of classical non-selective opioids in IBS therapy is limited to alleviating only the main symptoms. Additionally, the chronic administration of opioids is associated with the occurrence of severe adverse side effects. Therefore, a new idea has emerged to design synthetic opioids that interact simultaneously with the MOR, DOR, and/or KOR receptors to provide improved GI peristalsis and analgesic action with the elimination of adverse side effects. Therefore, biphalin as a MOR and DOR agonist seems to be a good candidate for this therapy (Table 6). The potential of biphalin in therapy for IBD and IBS was investigated by Fichna’s group.

Firstly, the effect of biphalin on an animal model of colitis (IBD) was studied by Sobczak M et al. [57]. Biphalin was injected i.c. and i.p. twice daily between day three and day seven at a dose of 5 mg/kg in mice with induced colitis by i.c. injection of TNB. The i.p. injection of biphalin had no effect on colitis, based on parameters such as the area of inflammation, MPO (myeloperoxidase) activity, or the ulcer score. On the other hand, biphalin injected directly into the colon (i.c. injection) showed a statistically significant decrease in the area of inflammation and ulceration and a minor decrease in the activity of MPO (Table 6). Moreover, i.p. and i.c injection of biphalin produced a strong analgesic effect by decreasing the number of mustard oil (MO)-induced pain-related behaviors in the hot plate test in the TNBS-treated mice mentioned in Section 2. Moreover, the time of rearing and jumping was delayed in the hot plate test in comparison with the vehicle-treated mice. These studies were continued by Zielińska M et al. [63], who characterized the effect of biphalin on mouse intestinal contractility in vitro and GI motility in vivo in animal models mimicking symptoms of diarrhea-predominant irritable bowel syndrome (IBS-D). The effect of biphalin on muscle contractility was characterized in the ileum and colon. The results showed that biphalin at a concentration of 10^−10^–10^−6^ M inhibited colonic and ileal smooth muscle contractions in a concentration-dependent manner. The effect was abolished by naloxone. In vivo, the anti-transit activity of biphalin was assessed by testing total gastrointestinal transit (WGT), colonic bead expulsion, fecal pellet output, and castor oil-diarrhea. In comparison with the control group, biphalin injected i.p. in a dose of 5 mg/kg significantly prolonged the WGT, inhibited colon GI hypermotility, and delayed the time of appearance of liquid feces after the oral administration of castor oil (mouse model of castor-induced diarrhea).

### 4.3. Biphalin as an Agent Improving Wound Healing

Pain often accompanies the healing of wounds. Opioid agonists are routinely used in the case of chronic wounds, burns, arthritis, and skin grafts to effectively reduce pain, especially inflammatory pain. Many studies have indicated that, depending on the concentration and route of application, an opioid agonist, such as morphine or fentanyl, may accelerate wound healing, not only by the modulation of the inflammatory phase but also by stimulating the formation of granulation tissue, collagen synthesis and angiogenesis, and the proliferation and migration of keratinocytes [85,86,87]. The activity of biphalin was also studied in this area, and the results of in vitro and in vivo research are presented in two publications (Table 6).

The main aim of Yildiz E et al. [64] was to evaluate the effect of biphalin on the proliferation and migration of primary human corneal epithelial cells (HCECs). Their studies showed that biphalin at a 1 μM concentration increased the number of cells passing through the membrane in a transwell migration assay and increased wound closure in a scratch assay, even though it did not stimulate the proliferation of HCECs. The partial inhibition of the effects of biphalin on HCACs migration by naloxone suggested the involvement of opioid receptors in this action. These authors were the first to show this effect of biphalin on the migration of HCACs; therefore, they proposed biphalin as an alternative analgesic agent which could improve the healing of post-surgical or post-traumatic corneal epithelial wounds.

The stimulating effect of biphalin on wound healing was observed by Muchowska et al. in 2020 [65]. In in vivo experiments, they demonstrated that topically applied biphalin, suspended in lanolin ointment at a 300 µL of 1 mM concentration, accelerated wound closure by about 30% in streptozotocin-induced rats compared to the negative control (streptozotocin-induced diabetic rats without any dressing, DM). Additionally, biphalin significantly improved the thicker epidermis and modulated the inflammatory phase by significantly increasing the number of macrophages in the wound tissue in comparison to the negative control. They also observed that in groups treated with biphalin, blood vessels showed a more typical round or oval shape with a well-marked lumen and larger perimeter than in the DM group, in which the neovascularization was uneven and aberrant.

### 4.4. Biphalin as a Neuroprotective Agent

Neuroprotection refers to mechanisms and strategies that protect neurons from degeneration and enables the recovery and restoration of their functions.

Most of the available studies indicate that all opioid receptor agonists could result in more effective neuroprotection than selective agonists that interact with only one type of opioid receptor. Therefore, biphalin as a non-selective opioid that simultaneously activates all opioid receptors could be a good candidate for a neuroprotective agent.

The first report on the neuroprotective potential of biphalin was published in 2011 [66] (Table 6). Kawalec et al. [66], using organotypic hippocampal cultures (OHC) challenged with NMDA, demonstrated that biphalin, co-administered with NMDA at the same time or with a delay of 0.5, 1, or 1.5 h, reduced NMDA-induced neuronal damage and the number dead cells by more than half. In these two cases, biphalin was used in much smaller doses than morphine, 0.025–0.1 μM vs. 3 μM, respectively. The neuroprotective effect of biphalin was abolished by naltrexone, which suggests the involvement of opioid receptors in this activity. Studies by Yang et al. in 2011 [67,68] and 2015 [69] were conducted in vitro, using mouse primary cortical neurons, and in vivo, using the two focal ischemia models: mouse transient or permanent middle cerebral artery occlusion models (tMCAO or pMCAO respectively) with or without reperfusion.

In an in vitro study, it was reported that biphalin at a 10 μM concentration [67] reduced edema in both hippocampal slices and primary neurons subjected to OGD (oxygen-glucose deprivation) [69], and also significantly decreased neuronal death and improved cell survival after challenging primary mouse cortical neurons with glutamate and hypoxia/aglycemia (H/A) [69]. In an in vivo study [67,68], the effects exerted by biphalin were greater than those observed for selective agonists such as DPDPE, DAMGO, and U50488. The edema and infarct ratios in mouse transient and permanent middle cerebral artery occlusion models were significantly decreased after the i.p. injection of biphalin. The administration of biphalin also affected decreased neurodegeneration in hippocampal, cortical, and striatal brain tissue after ischemia compared to the vehicle-treated group. The results obtained in 2011 [68] suggest that the reduction in neuronal cellular edema is caused by the modulation of ion transporter function. Biphalin significantly decreased the expression of Na^+^, K^+^, 2Cl^−^ cotransporter (NKCC) and the translocation of the conventional isoforms of protein kinase C (PKC). All effects were reversed by naltrexone, indicating that the protective effect of biphalin is mediated through opioid receptors. Furthermore, the results obtained by Yang L et al. in 2015 [69] also showed the antioxidative properties of biphalin. Biphalin at a 10 nM concentration significantly reduced ROS generation compared to untreated cells and cells treated by selective agonists. In this case, however, the effect was partially reversed by naltrexone, suggesting that this effect may be mediated through a non-opioid receptor mechanism [69]. Independently, the antioxidative properties of biphalin were studied by Garbuz et al. [88]. They demonstrated that the antioxidative capacity of biphalin (IC_50_ = 8 μM) was 3.6 times greater than that of ascorbic acid (IC_50_ = 29 μM) and over ten times greater than that of morphine (IC_50_ = 81 µM). The results were obtained using ABTS in a “tube test” without the presence of receptor proteins, which confirms the earlier observation [69] that the antioxidative effect of biphalin could be related not only to the opioid receptors.

In another study, the neuroprotective activity of biphalin was also tested by Lipkowski’s group. It was found that biphalin protects against cognitive deficits in a mouse model of mild traumatic brain injury (mTBI) (Table 6) [70]. Biphalin reduced cortical and hippocampal neurodegeneration, as shown by silver staining. The data indicated that opioid receptor activation by biphalin may provide neuroprotection against post-traumatic neurodegeneration processes and may protect against memory impairments. These findings corroborated the early findings on the neuroprotective properties of biphalin and described its effects from a behavioral perspective.

Furthermore, the neuroprotective effects of biphalin were also investigated in a mouse model of neonatal hypoxia–ischemia (HI) brain injury [71]. Biphalin, in doses of 5, 10, or 20 mg/kg, was injected intraperitoneally to mouse pups immediately after HI (1h hypoxia, 10% O_2_ in N_2_). To determine the underlying mechanism, the opioid antagonist naloxone or phosphatidylinositol-3-kinase inhibitor Ly294002 was administered. Infarct volume, brain edema, phosphorylated Akt, and apoptosis-related protein levels were evaluated by using a combination of 2,3,5-triphenyltetrazolium chloride staining, brain water content, and Western blotting 24 h after HI. The results showed that biphalin, at a dose of 10 mg/kg, significantly reduced the infarct volume and ameliorated brain edema. Biphalin also showed long-term protective effects against the loss of ipsilateral brain tissue and resulted in improvements in neurobehavioral outcomes. Moreover, biphalin treatment significantly preserved phosphorylated Akt expression, increased Bcl-2 levels, decreased Bax levels, and cleaved caspase 3 levels after HI. In all the experiments, the neuroprotective effect was reversed by naloxone and Ly294002, respectively, which allowed the authors to conclude that biphalin protects against HI brain injury in neonatal mice through the activation of the opioid receptor/phosphatidylinositol-3-kinase/Akt signaling pathway.

### 4.5. Cardiorespiratory Effect of Biphalin

The influence of biphalin on respiratory patterns and blood pressure has been studied by Wojciechowski et al. (Table 6). The authors tried to answer the question of how the activation of peripheral opioid receptors by biphalin influences cardiorespiratory functions [72] and whether the DOR receptor is involved in the cardiorespiratory effects of the systemic injection of biphalin [73]. In a 2009 study [72], biphalin was administered intravenously to urethane–chloralose-anesthetized adult Wistar rats. The results showed that biphalin challenging depresses respiration by evoking a significant increase in tidal volume (Vt) from the immediate post-apnoeic phase to later time points, and, at the same time, produces a significant reduction in respiratory rate. In another work, Wojciechowski et al. [73] noted that the activation of the DOR receptor contributes to the depressive response produced by biphalin.

The hypotensive effect of biphalin in spontaneously hypertensive rats (SHR), as well as other hypertension models essentially different from SHR, was studied by Sadowski’s group [74]. The first model was developed by the exposure of normotensive rats without right kidneys to high salt intake (HS/UNX). In the other model, hypertension was obtained by the chronic infusion of Ang II (Ang-iH).

In a short paper, Bądzyńska et al. [74] reported results suggesting that biphalin might find therapeutic applications for stress-induced hypertension in humans. They showed that biphalin, at a dose of 150 µg/kg/h, significantly decreased mean arterial pressure (MAP) from 143 to 130 and from 177 to 167 mmHg, while the heart rate (HR) was slightly decreased. The renal blood flow (RBF) was modestly increased, but renal and hind limb vascular resistance were significantly decreased.

The aim of another study by Bądzyńska [75] was to examine the hypotensive and cardiovascular effects of biphalin in two other hypertension models, HS/UNX and Ang-iH (Table 6). The experiments were performed using anesthetized and conscious rats. Similar to the previously described publication, the MAP, RBF, vascular resistance, and also iliac blood flows (IBF) were measured before and after i.v. infused biphalin at 300 μg/kg. The results showed that biphalin, in anesthetized and conscious SHR rats, significantly decreased the MAP by 10 and 20 mmHg, respectively. In anesthetized HS/UNX rats, the MAP increased by ~6–7 mmHg; without anesthesia, only transient decreases occurred, and a similar effect was observed in normotensive rats. Biphalin was not shown to change the MAP in Angi-iH rats. In contrast to biphalin, the responses in MAP, HR, RBF, IBF, and IVR exerted by intravenous morphine at 1.5 mg/kg in rat models were often delayed.

The novel findings of these studies were that the hypotensive action of intravenous biphalin is observed in both anesthetized and conscious SHR but in not two other essentially different models of rat hypertension or in normotensive Sprague-Dawley controls. These results indicate that the success or failure of an antihypertensive treatment regime may critically depend on the pathogenesis of hypertension. However, the authors believe that the main advantage of biphalin or biphalin-like drugs as a treatment in antihypertensive therapy would be that they do not endanger renal perfusion.

## 5. Conclusions

For many years, biphalin has continued to be a source of inspiration for many scientists. Every year, at least a few new biphalin analogs have been designed, synthesized, and tested [27,89,90], and their non-analgesic properties related to the opioid system are still being discovered and studied [42,65,71]. This comprehensive review shows how many areas biphalin has been studied in. Extensive research on the antinociceptive effects of biphalin has shown its excellent properties in acute, neuropathic, and chronic pain and that it is 1000 times more potent than morphine when administered intrathecally. It was found that biphalin exhibited excellent analgesic properties in many different animal pain models, such as skin cancer (melanoma) pain, inflammatory mustard-oil induced pain, a neuropathic pain model of a chronic construction injury (CCI), and acute thermal pain. Moreover, biphalin, while having excellent analgesic properties, causes fewer side effects than the more commonly used analgesic drugs such as morphine or fentanyl. Considering the presence of opioid receptors on the surface of many cells and tissues, it is understandable that researchers are interested in studying the additional properties of biphalin, rather than only its analgesic effects.

The biological properties of biphalin have been studied for almost 40 years, and the results of these studies indicate its very beneficial biomedical activity. Biphalin exhibited antiviral properties in an FLV-infected Mus dunni cells model, anticancer properties by the inhibition of human glioblastoma T98G cell growth, and wound healing-improvement properties via the increased migration of corneal epithelial cell cultures. Biphalin’s neuroprotective activity was verified using hippocampal organotypic culture, OGD-exposed mouse primary cortical neurons, in vivo in mild traumatic brain injury, and tMCAO and pMCAO models. The immunomodulatory activity of biphalin was confirmed in vitro in various LPS-induced immune cell cultures, such as lymphocyte T, NK cells, macrophage, and in vivo in TNBS-induced colitis. It is known that chronic pain is always associated with inflammation and it was shown that biphalin may also be a great antinociceptive agent in the therapy of pain in inflammatory diseases. Furthermore, biphalin affects many physiological functions such as cardiorespiratory functions, blood pressure, and renal flows.

Notwithstanding, so far no drug has been developed based on the biphalin structure that could be used in a clinic. We are convinced that further research on biphalin will provide a better understanding of its biological effects. As a non-selective opioid receptor agonist, biphalin could become a panacea for many opioid system-dependent physiological dysfunctions.

## Figures and Tables

**Table 1:** Binding affinity of biphalin for the MOR, DOR, and KOR receptors, determined in various laboratories, by competitive displacement of the appropriate selective radioligands (given in the footnotes to the table) using different membranes.

Source of the Opioid Receptors	K_i_ (nM) ± S.E.M. *			IC_50_ (nM) ± S.E.M. *			Ref.
	MOR	DOR	KOR	MOR	DOR	KOR	
Guinea pig brain membrane	12 ± 2 ^a^	4.6 ± 0.2 ^b^	270 ± 15 ^c^				[16,17]
Rat brain membrane				1.4 ± 0.4 ^d^	2.6 ± 0.3 ^e^		[18]
Rat brain membrane				0.74 ± 0.26 ^d^	2.96 ± 0.22 ^e^	35.1 ± 2.0 ^f^	[19]
Guinea pig brain homogenate				2.8 ± 0.4 ^d^	5.2 ± 0.3 ^g^		[20]
Rat brain homogenate	0.19 (0.12-0.29) ^h^ (95% CI) *	1.04 (0.69-1.55) ^i^ (95% CI) *					[21]
Rat brain membrane	0.79 ^h^	3.5 ^j^					[22]
Rat brain membrane	2.6 ± 0.7 ^h^	15 ± 2.3 ^k^	283.1 ± 18 ^f^				[23]
CHO cell transfected with cloned human δ-opioid receptor membrane		46.5 ± 1.5 ^l^					[24]

* Ki—inhibitory constants; IC_50_—the half-maximal inhibitory concentration; S.E.M.—standard error of the mean; 95% CI—95% confidence interval. ^a^ [^3^H]naloxone; ^b^ [^3^H] DADLE (H-Tyr-D-Ala-Gly-Phe-D-Leu-OH); ^c^ [^3^H]EKC (ethylketazocine); ^d^ [^3^H]CTOP (D-Phe-c[Cys-Tyr-D-Trp-Orn-Thr-Pen]-Thr-NH_2_); ^e^ [^3^H][p-ClPhe4]DPDPE (H-Tyr-c[d-Pen-Gly-Phe(4-Cl)-d-Pen]-OH); ^f^ [^3^H]U-69,593; ^g^ [^3^H]DPDPE (H-Tyr-c[d-Pen-Gly-Phe-d-Pen]-OH); ^h^ [^3^H]DAMGO (H-Tyr-D-Ala-Gly-*N*-MePhe-Gly-ol); ^i^ [^3^H]DELT II (H-Tyr-D-Ala-Phe-Glu-Val-Val-Gly-NH_2_); ^j^ [^3^H][Ile]DELT II (H-Tyr-Ile-Phe-Glu-Val-Val-Gly-NH_2_); ^k^ [^3^H]Ile^5,6^ deltorphin II (H-Tyr-Ala-Phe-Glu-Ile-Ile-Gly-NH_2_); ^l^ [^3^H]naltrindole.

**Table 2:** Biphalin and deltrophin II [^35^S] GTPγS binding properties (G-protein activation).

Compound	DOR		MOR		KOR		Ref.
	E_max_ ± S.E.M * (%)	EC_50_ ± S.E.M * (nM)	E_max_ ± S.E.M * (%)	EC_50_ ± S.E.M * (nM)	E_max_ ± S.E.M * (%)	EC_50_ ± S.E.M * (nM)	
Biphalin			176.9 ± 4.9	75.4			[22]
	219.6 ± 5.7	90.5 ± 25	178.2 ± 3.6	12 ± 4.6	108.9 ± 4.1	Amb ^#^	[23]
	98 ± 10	34.0 ± 13.1					[24]
	83 ± 4	1.1					[35]
	27 ± 3.5	2.5 ± 0.5	25 ± 4.7	6 ± 0.2			[34]
			238 ± 4.9	pEC_50_ ± S.E.M * −7.0 ± 0.08			[21]
Deltrophin II	96 ± 2	9.3 ± 4.2					[24]

^#^ Amb.—ambiguous fitting since the compound did not stimulate the receptor above basal activity significantly; * E_max_—maximum stimulatory effect; EC_50_—half-maximal effective concentration; S.E.M.—standard error of the mean; pEC_50_—negative logarithm of EC_50_.

**Table 3:** Results of biphalin functional tests at opioid receptors as determined by various laboratories.

Compound	Bioassay IC_50_ (nM) ± S.E.M *		Ref.
	GPI	MVD	
Biphalin	1.94 ± 0.29		[9]
	8.8	27	[25]
	8.8 ± 0.3	27 ± 1.5	[35,36]
D-Ala^2^-Met-enkephalinamide	22.8 ± 3.4		[9]

* IC_50_—the half maximal inhibitory concentration; S.E.M.—standard error of the mean.

**Table 4:** Analgesic activity of biphalin in an acute pain animal model. Data were obtained from various laboratories.

Pain Model/Animal/Test	Route of Administration/ Dose	Effect	Ref.
Mouse, hot-plate assay	i.p./ 5, 10, 20 mg/kg	The dose of 20 mg/kg increased the response latency 60 min after the injection by 185.8% compared to the pre-injection control.	[9]
Rat, hot plate test	i.p./ 10, 20 mg/kg	The dose of 20 mg/kg increased the latency of the response 60 min after administration by 177.4% of the pre-injection control value.	[37]
Rat, tail-flick test Rat, tail pinch test	s.c./2.5, 5, 10, 40, 80 μmol/kg i.v./10, 20, 40 μmol/kg i.t./0.5, 10, 20 nmol	s.c.—the ED_50_ (95% Cl) was 7.88 nmol/kg (6.33–9.81) for tail flick and 5.58 nmol/kg (4.80–6.48) for tail pinch. i.v.—the ED_50_ (95% Cl) were 17.87µmol/kg (15.06–21.19) for tail flick and 18.9 µmol/kg (15.0–23.8) for tail pinch test. i.t.—ED_50_ (95% Cl) were 2.88 nmol (1.09–7.54) for tail flick and greater than 250 nmol for tail pinch.	[48]
Non burned (NB) rat, Burned (B) rat, tail-flick test.	i.v./ NB group: 5, 10, 15 μmol/kg B group: 5, 7.5, 10 μmol/kg	ED_50_ (95% Cl) are 7.34 (6.37–8.30) and 10.69 (8.66–12.73) μmol/kg in the B and NB groups, respectively.	[49]
Rat, tail flick test	i.v./5 μmol/kg	~45% MPE at 5 min after administration.	[50]
Rat, tail-flick test	i.t./0.75, 2.5, 5.0 μg	~40% MPE at a dose of 0.75 μg at 15 min after administration. ~50% MPE at a dose of 2.5 μg at 15 min after administration. ~80% MPE at a dose of 5 μg at 30 min after administration.	[51]
ICR mice tail flick test	i.c.v./1, 3, 10, 30, 100 pmol/mouse i.t./8.8, 880, 8800 pmol/mouse i.p./2.6, 4, 5.3, 8.8 μmol/kg	i.c.v.—A_50_ (95% Cl) of 4.9 (1.6–15.3) pmol/mouse and a time to peak effect of 20 min. i.t.—~60% antinociceptive response up to a dose of 8.8 nmol/mouse. i.p.—A_50_ (95% Cl) of 5.7 (3.7–8.7) μmol/kg; the peak effect after 20 min.	[47]
ICR mice, tail-flick test	i.c.v./0.4 nmol/kg i.v./685 nmol/kg	i.c.v.—68% MPE at 20 min then quickly dropped to <10% MPE by 45 min. i.v.—83% MPE at 30–60 min.	[52]
Rat, tail-flick test	i.v./150, 300, 600, 1200 nmol/kg i.m./4300, 8600, 17200 nmol/kg s.c./4300, 8600, 17200 nmol/kg	i.v.—A_50_ ± S.E.M 523 ± 9 nmol/kg. i.m.—A_50_ ± S.E.M 236 ± 42 nmol/kg. s.c.—A_50_ ± S.E.M 9276 ± 1290 nmol/kg.	
Rat with encephalomyelitis (EAE)	i.v.	83% MPE at 15 min after administration. The analgesic potency correlated well with the progression of EAE	[43]
Rat, tail-flick test	i.t./0.001, 0.005, 0.0125, 0.025, 0.5, 2, 20 nmol	60–70% MPE at a dose of 0.005 nmol at 15 min after administration. 100% MPE at doses of 0.5 and 2.0 nmol at 15–30 min after administration. At a dose of 20 nmol, long-lasting analgesia, body rigidity.	[53]
Rat, tail-flick test	i.t./0.005 μmol	60–70% MPE at 15 min after administration.	[54]
Mouse, hot plate test	i.c.v./0.1 nmol/mouse i.v./1500 nmol/kg	i.c.v.—~90% of MPE at 30 min after administration. i.v.—~40% of MPE at 30 min after administration.	[23]
Mouse, tail-flick test	i.c.v./0.1 nmol/mouse i.v./1500 nmol/kg	i.c.v.—~85% of MPE at 15–45 min after administration. i.v.—~40% of MPE at 30 min after administration.	
Rat, hot-plate test	i.c.v./1 nmol/kg i.v./1200 nmol/kg	i.c.v.—71% MPE at 30 min after administration. i.v.—68.32% MPE at 45 min after administration.	[55]
Mouse, tail-flick test	i.t./0.01 nmol/animal i.c.v/0.01 nmol/animal	~80% of MPE were obtained at 15 min after injection in both tests i.t. and i.c.v.	[22]

ED_50_—median effective dose; 95% CI—95% confidence interval; MPE—maximal possible effect; A_50_—dose producing a 50% antinociceptive effect; S.E.M.—standard error of the mean.

**Table 5:** Analgesic activity of biphalin in different animal pain models.

Pain Model/Animal/Test	Route of Administration/Dose	Effect	Ref.
Cancer pain/Mouse, paw withdrawal test, tail-flick test	i.v./5, 10, 15, 20 μmol/kg	Dose-dependent increase in the total analgesic effect, higher doses caused motor impairments and muscle rigidity. The complete alleviation of thermally-induced pain was observed for a dose of 20 μmol/kg, %MPE reached 100% in most mice. At a dose of 20 μmol/kg, a strong peak analgesic effect in the tumor-bearing paw, %MPE: 55.5 ± 4.5. ED_50_ (μmol/kg, 95% CI) for: tumor-bearing paw: 19.10 (18.0–20.2); intact paw: 17.6 (15.6–18.7); tail-flick: 11.8 (10.9–12.6).	[21]
Acute and inflammatory pain/Mouse, formalin test	s.c./0.1 nmol/animal	Reduced formalin-induced pain behavior both in the early (acute pain) and in the late phase (inflammatory pain) of the test.	[22]
Neuropathic pain/Rat, mechanical and thermal hypersensitivity as measured by von Frey and cold plate tests.	i.t./20, 200, 1000 µM	Attenuated the development of tactile hypersensitivity as measured by von Frey test 30 min after drug injection, as compared to the vehicle-treated CCI (chronic constriction injury)-exposed rats (12.78 g ±0.55 versus 19.88 g ± 0.63, 25.58 g ± 0.32, and 25.91 g ± 0.09). Attenuated the development of thermal hypersensitivity as measured by cold plate test 35 min after drug administration, as compared to the vehicle-treated CCI-exposed animals (6.93 s ± 2.97 versus 20.11 s ± 2.81, 26.27 s ± 1.67, and 29.90 s ± 0.11, respectively, for administered doses).	[56]
Visceral pain/Mouse with acute colitis, colonic inflammation, mustard oil-induced pain responses and hot plate test	i.p./5 mg/kg i.c./5 mg/kg	Produced a strong analgesic effect in inflamed mice (mustard oil-induced pain) after i.p. injection (10 ± 1 vs. 51 ± 8 number of pain responses for vehicle-treated mice) and after i.c. injection. Delay in rearing (91 ± 12 vs. 48 ± 4 for biphalin vs. vehicle-treated mice) and jumping (183 ± 17 vs. 83 ± 13, respectively) times in the hot plate test.	[57]

%MPE—% of maximal possible effect; ED_50_—median effective dose; 95% CI—95% confidence interval.

**Table 6:** Other activity of biphalin.

Activity	Cell Line/Animal Model	Route of Administration/Dose	Effect	Ref.
Antiviral	Mus Dunni cells infected with FLV/ in vitro	10^−6^–10^−8^ M	Inhibition of FLV RT activity.	[59,60]
		15–30 µg mixed with 0.5 ng/mL of AZT	Inhibition of FLV replication by 50%.	
		100 μg/mL mixed with 10^−6^ of splenocytes	Inhibition of FLV replication by 58%.	
		100 pg/mL mixed with 1 ng/mL of AZT and 10^−6^ of splenocytes	Inhibition of FLV replication by 68%.	
		50 μg/mL mixed with 250 ng of INF-γ	Inhibition of FLV RT activity by 94%.	
Antiproliferative (anticancer)	Human glioblastoma T98G/in vitro	50 nM–40 μM	Inhibition of tumor cell growth and decrease in proliferation rate. Decline of cell ability to form colonies. Modulation of Ki69 proliferation index.	[61]
Immunomodulatory	Lymphocyte T, NK cells, suspension of human PMBCs and mouse macrophage RAW 264.7/in vitro	10^−8^ or 10^−10^ M	Increase in cytotoxicity of NK cells. Stimulation of lymphocyte T proliferation. Increase in IL-2 production. Increase in chemotactic activity of monocytes. Marginal decrease in TNF-α and NO production by LPS.	[62]
	Microglia cell culture LPS-stimulated/in vitro	10 µM	Decrease in NO production, expression of Iba1, iNOS, IL-1β, IL-18, IL-6, IL-10, TNFα, pSTAT3, pERK1/2, p-NF-κB, p-IκB, p-p38MAPK, TRIF, and upregulation of SOCS3, TLR4, MyD88.	[56]
	CCI, chronic construction injury model of neuropathic pain in *Wistar* rats/in vivo	i.t./20, 200, 1000 μM	Diminished symptoms of neuropathy in von Frey test and cold plate test.	
	Semi-chronic colitis model in *Balb/C* mice/in vivo	i.c./5 mg/kg	Decrease in macroscopic and ulcer scores.	[57]
		i.p./5 mg/kg	No noticeable effect on colitis.	
	Ileum and distal colon from *Balb/C* mice/in vitro	10^−10^–10^−6^ M into organs baths	Inhibition of colonic and ileal smooth muscle contractions.	[63]
	*Balb/C* mice/in vivo	i.p./5 mg/kg	Inhibition of colon motility. Prolongation of GI-transit and inhibition of colonic bead expulsion. Reversed hypermotility and exertion of anti-diarrheal effect.	
Wound healing	Corneal epithelial cell culture (HCEC)/in vitro	1 μM, 10 μM	Increased wound closure in in vitro wound healing model and increase in cell migration in transwell migration assay.	[64]
	Streptozotocin-induced diabetic *Wistar* rats/in vivo	1 mM	Reduction in wound size by 77% after 14 days of healing. Increase in the number of macrophages on day 4. Increase in the thickness of the epidermis on day 21.	[65]
Neuroprotective	Hippocampal organotypic culture/in vitro	0.025–0.1 μM	Reduction in NMDA-induced neuronal damage. Reduction in the number of dead cells.	[66]
	Mouse primary cortical neurons exposed to OGD/in vitro	0.001 nM–1 nM	Decrease in cell volume after OGD treatment.	[67]
	Hippocampal slices exposed to OGD/in situ	0.01 µM–10 µM	Decrease in water content compared with selective agonists.	
	pMCAO model of *CD-1* male mice/in vivo	i.p./5.7 µmol/kg	Decrease in edema (53%) and infarct ratios (48%) and neuronal recovery from stroke.	
	Mouse primary cortical neurons OGD treatment/in vitro	0.01 nM	Decrease in cell volume after OGD treatment.	[68]
	tMCAO and pMCAO model of *CD-1* male mice/in vivo	i.p./5.7 μmol/kg	Decrease in edema ratios by 66.6% tMCAO and by 58.3% pMCAO; decreased infarct ratios by 52.2% tMCAO and by 56.4% pMCAO. Improvement of neurological scores and locomotor activity. Decrease in penumbral expression of Na^+^, K^+^, 2Cl^−^ cotransporter and translocation of isoforms of protein kinase C.	
	Mouse primary cortical neurons challenged with glutamate and hypoxic/aglycemic (H/A)/in vitro	10 nM	Decrease in neuronal death; decrease in ROS production.	[69]
	tMCAO with reperfusion/in vivo	i.p./5 mg/kg	Reduction in the edema ratios by 76.4% and reduction in the infarct ratio by 77.3%.	
	Mouse model of mild traumatic brain injury (mTBI)/in vivo	i.v./10 mg/kg	Improvement of recognition memory in mTBI mice. Only a partial reversion of depressive-like immobility in mTBI mice; failed to reverse spatial memory deficits in mTBI mice. Immediate, delayed, or chronic biphalin administration improved spatial memory in mTBI mice.	[70]
	Mouse neonatal HI model/in vitro	i.p./5, 10, 20 mg/kg immediately after HI	Reduction in the infarct volume, brain edema, and brain atrophy. Improvement of neurobehavioral outcomes in neonatal HI in mice. Reduction in the infarct volume and brain edema required the activation of the opioid receptor/PI3K/Akt signaling pathway. Inhibition of HI-induced brain atrophy after long-term survival. Regulation in the expression level of phosphorylated Akt and apoptotic proteins after HI.	[71]
Cardiorespiratory	*Wistar* rats/in vivo	i.v./0.3 mg/kg	Evocation of apnoea with a mean duration of 13.5 ± 1.25 s. Evocation of significant increases in Vt from immediate post-apnoeic phase to later time points. Reduction in respiratory rate. Decrease in breathing rate and increase in tidal volume, hypotension, and bradycardia.	[72,73]
Blood pressure and renal flows	Spontaneously hypertensive rats (SHR) Normotensive *Wistar-Kyoto* rats (WKY)/in vitro	i.v./150 μg/kg/h	Decrease in mean arterial blood pressure (MAP). Modest increase in renal blood flow and decrease in renal and hind limb vascular resistance.	[74]
	Normotensive S-D rats Spontaneously hypertensive rats (SHR) Male S-D rats on a high-salt diet (HS/UNX) Male S-D rats with f angiotensin-induced hypertension (Ang-iH)/in vivo	i.v./300 µg/kg/h	Decrease in blood pressure in SHR but not in the HS/UNX and Ang-iH or normotensive WKY and S-D rats. In anesthetized and conscious SHR, decrease in MAP by ~10 and ~20 mmHg, respectively. In anesthetized HS/UNX and normotensive rats, increase in MAP by~6–7 mmHg. No changes in the MAP of Ang-iH rats.	[75]
